# A systematic review and meta-analysis of studies on screening for mild cognitive impairment in primary healthcare

**DOI:** 10.1186/s12888-022-03730-8

**Published:** 2022-02-09

**Authors:** Leila Karimi, Alireza Mahboub–Ahari, Leila Jahangiry, Homayoun Sadeghi-Bazargani, Mostafa Farahbakhsh

**Affiliations:** 1 Health Education and Health Promotion Department, School of Public Health, Tabriz University of Medical Sciences, Tabriz, Iran; 2 School of Management and Medical Informatics, Tabriz Health Services Management Research Center, Tabriz University of Medical Sciences, Tabriz, Iran; 3 Research Center for Evidence Based Medicine, Tabriz University of Medical Sciences, Tabriz, Iran; 4 Road Traffic Injury Research Center, Tabriz University of Medical Sciences, Tabriz, Iran; 5 Psychiatrics Research Center, Tabriz University of Medical Sciences, Tabriz, Iran

**Keywords:** Early diagnosis, Dementia, Mild cognitive impairment, Primary health care, Screening

## Abstract

**Background:**

Cognitive disorders and dementia have an important effect on individual independence and orientation. According to the Alzheimer's Disease International (ADI) 75% of people with dementia are not diagnosed; this may be as high as 90% in some low- and middle-income countries. This systematic review and meta-analysis aimed to identify the test performance of screening tools and compare them pairwise. The findings of our study can support countries in planning to establish and care for mild cognitive impairment in primary health centers.

**Methods:**

Medline (PubMed), Scopus, Cochrane, Dare, All EBM Reviews, CRD (OVID), and Proquest were searched from 2012 to November 2021. The risk of bias was assessed through the QUADAS-2 instrument. Given the high heterogeneity between studies, a random-effects model was used to calculate the pooled effect sizes for diagnostic accuracy measures (sensitivity, specificity, and area under curve indices). *I*^*2*^ test was used for assessing heterogeneity and predefined subgroup analyses were performed using participants’ age, country’s income, and sample size of studies.

**Results:**

A systematic search identified 18,132 records, of which, 20 studies were included in the quality assessment, and six were included in quantitative analysis. None of the studies had examined the feasibility or efficiency of mass screening. According to a pairwise comparison, IQCODE, AD8 and GPCOG showed equal or better diagnostic performance relative to the MMSE in terms of sensitivity and specificity. The random-effect model for the MMSE showed the pooled sensitivity equal to 0.73 (95% CI 0.57–0.90), the pooled specificity equal to 0.83 (95% CI 0.75—0.90), and the pooled AUC equal to 0.88 (95% CI 0.83–0.93).

**Conclusion:**

Several benefits have been attached to short tests making them a suitable choice for use in primary healthcare settings. Considering factors such as accuracy, time of application, ease of scoring, and utilization charges, tests such as IQCODE, AD8, and GPCOG or appropriate combination with counterpart tools seem to be good alternatives to the use of the MMSE in primary care.

## Background

Cognitive disorders and dementia have an important effect on individual independence and orientation. Alzheimer’s is characterized by impaired memory and dysfunction; it is one of the areas of aphasia, apraxia, amnesia, and dysfunction, which has a significant impact on individual and social functioning [1]. According to the World Alzheimer Report, over 55 million people worldwide live with dementia, and this number is expected to increase to 78 million by 2030. According to the mentioned report, 75% of people with dementia are not diagnosed; this may be as high as 90% in some low- and middle-income countries [2].

Additional research by the National Institute on Aging (NIA) at the National Institutes of Health (NIH) and the Alzheimer's Association (NIA-AA) highlighted modernizing concept in Alzheimer’s disease diagnosis [3]. The research groups introduced Alzheimer’s disease in a continuum with three discrete phases including preclinical, Mild Cognitive Impairment (MCI), and dementia. They suggested that Alzheimer’s disease (AD) is a pathophysiological construct similar to other diseases such as diabetes and osteoporosis. By using biomarkers, a clinical specialist might detect the disease in a person based on symptoms [4]. However, physicians are less likely to be able to diagnose cognitive disorders by formal examining or performing daily visits [5], therefore, up to 76% of patients are diagnosed only in moderate or severe dementia [6–8]. Early diagnosis of cognitive impairment can give patients and their families the opportunity to receive care in the early stages of the disease; this will lead to a better prognosis and improve living standards. Although early detection of cognitive impairment cannot halt the onset of the disorder, and existing treatments cannot reverse the course of the disease, the health, psychological, and social benefits of early detection are important enough to make a screening program worthwhile [9]. Werner et al. [10] conducted a systematic review to investigate dementia diagnosis disclosure among the patients and their families. Based on their findings, most studies have been positive about the disclosure of the disease. The patients' families have acknowledged that they were initially skeptical about the disease disclosure, then they later adapted it. Awareness of the diagnosis has led to better planning and preparation for the future.

There has been a growing interest among researchers and health systems for the early identification of people at risk of developing dementia. In fact, early accurate diagnosis of AD is a major global health priority [11]. The global action plan of the World Health Organization (WHO) on the public health response to dementia targets at least 50% of countries to diagnose 50% of the estimated number of people with dementia by 2025 [2]. The US Preventive Services Task Force (USPSTF) in its last update, reported that there was insufficient published evidence of better clinical outcomes as a result of routine screening for cognitive impairment in older adults. However, the Task Force recognized that the use of cognitive assessment tools can increase the detection of cognitive impairment [12]. Subsequently, the Patient Protection and Affordable Care Act (PPACA) in the United States recommended early diagnosis of cognitive impairment during the annual wellness visit. The workgroup developed ten recommendations for improving the early detection and care for dementia, concerning the implementation of cognitive screening practice in personalized healthcare [13]. According to the principals of Annals Wellness Visits (AWV), the early detection process is likely to occur in a primary care setting by using brief screening tests (taking a minimum time to administer), used by non-physician practitioners. Therefore, it is necessary to have easy-to-score, quick, open access, and sensitive tests to identify people with dementia in primary healthcare [14]. In recent years, systematic reviews and meta-analyses have attempted to identify diagnostic accuracy of both comprehensive and brief instruments for cognitive impairment and Alzheimer’s [15, 16]. Most of them have examined cognitive screening measures in secondary or tertiary care settings where the practice is run by physicians or neuropsychologist experts. The test performance of screening tools has not been widely assessed in the literature. In the study by Pelegrini et al. [15], diagnostic strategies in primary healthcare settings have been examined across low and middle-income countries. In spite of the short time interval of literature search (2013 to 2018), the study has only reported a sort of diagnostic criteria for screening tests’ performance and compared it among countries from different income streams. However, the gap of suitable instruments for use in primary healthcare settings has still been remained questionable. Lin et al., in an updated systematic review, attempted to address the benefits, harms, and diagnostic accuracy of brief screening instruments to detect cognitive impairment in community -dwelling older adults [16]. In spite of their conclusion in favor of the benefits of using brief instruments, they have not recognized empirical evidence on screening to improve decision-making. Considering the importance of early diagnosis for cognitive impairment as well as the consensus on primary care setting as the best start setting for assessment, our systematic review and meta-analysis aimed to identify test performance of screening tools and compare them pairwise. The findings of our study can support countries in planning to establish dementia care in primary health care centers.

## Methods

The present systematic review was conducted in accordance with the preferred report items for systematic review and meta-analysis studies (PRISMA) [17]. The systematic review protocol was registered in the International Prospective Register of Systematic Reviews (PROSPERO) database with the code CRD42020156638.

### Inclusion and exclusion criteria

All English original studies including a) screening early detection of cognitive disorders in a primary care setting, b) using short questionnaires (according to the Alzheimer Association, the questionnaires that take less than 5 min to administer), c) and reporting sensitivity, specificity, positive and negative predictive values, and AUC measures for diagnostic tests and d) screening mild dementia were searched. The exclusion criteria were: a) studies that only examined the characteristics of diagnostic methods, b) or evaluated patient or provider’s opinion about the instruments, c) studies applied laboratory markers or imaging techniques to diagnose a particular type of dementia or Alzheimer's disease.

### Data sources and search strategy

Databases including Medline (PubMed), Scopus, Cochrane, Dare, All EBM Reviews, Center for Research and Dissemination (CRD) via OVID, and Proquest were searched from the beginning of 2012 to November 2021. A search strategy is presented below for PubMed. A supplementary search across the references list and citations of included studies were also performed in Google Scholar to find related articles.

(TITLE-ABS-KEY (dementia OR Alzheimer OR "Cognitive Disorders" OR "Cognitive impairment" OR "Cognition Disorders" OR "cognitive decline" OR "cognitive loss") AND TITLE-ABS-KEY (screening OR "Early detection" OR "early diagnosis")) AND PUBYEAR > 2012 AND PUBYEAR < 2021.

### Selection of studies

The study selection was independently done by two authors (LK and LJ). Any disagreement was resolved by the systematic review consultant (HS) or the clinical consultant (MF). After eliminating duplicates in the reference management software (EndNote) and manually (sorting by the title and year of the study), the titles and abstracts of the studies were screened according to the inclusion criteria. At this stage, screening programs were identified and studies that met the exclusion criteria were excluded. For the studies without the original article, the authors contacted the corresponding author (send an email or message in www.researchgate.net). If the reply message was not received after sending the message, the article was removed.

### Data extraction

An Excel form was designed by the research team then administered to gather information about the author, year, country, population and place of the study, sample size, index, and reference test, reported outcome, and cut-off point. Data were independently extracted by (LK) and (AM) and sent to the (LJ) step by step for review and approval.

### Risk of bias and quality assessment

In order to assess the risk of bias in the studies, The Quality Assessment of Diagnostic Accuracy Studies-2 (QUADAS-2) tools were used [17]. This tool has four domains of patient selection (three questions), index test (two questions), reference test (two questions), flow, and time (four questions). The probability of bias existence is reported in three levels of bias: low, uncertain, and high. Concerns about the usability of each domain are also reported in three forms: low, high, and unspecified. In fact, the purpose of this question is to evaluate the ability of the domain to answer the research question. In order to evaluate the quality of the studies, a software program designed by the QUADAS group was used. In this program, questions of each domain are listed, which by entering studies and evaluating them, the program allows the researcher to produce graphs and evaluation results in the form of excel tables. The risk of bias was assessed by LK and AM. In cases where clinical or epidemiological consultation was required, cases were raised and resolved with consulting professors (HS and MF). For minimizing biases and increasing reliability, selecting the studies for this systematic review was conducted through dual revision by two researchers. Cohen’s Kapa coefficient statistic was used for reporting the agreement.

### Outcome measurement criteria

The outcome of interest consisted of the diagnostic accuracy indices of the screening tests, including sensitivity, specificity, or data that could be used to derive these values.

### Summary of study findings and statistical analysis

In order to evaluate the accuracy of diagnostic screening tools, sensitivity and specificity of indices and reference tests were compared and reported in terms of study number and sample size. Given the high heterogeneity between studies, a random-effects model was used to calculate the pooled specificity, sensitivity, and AUC. *I*^*2*^ test was used for assessing heterogeneity and predefined subgroup analyses were performed using participants’ age, country’s income, and sample size of studies. The data were analyzed using STATA version 14 (STATA Corp, College Station, TX, USA). P-values of less than 0.05 were considered statistically significant. Publication bias test was conducted by funnel plot analysis*.*

### Ethical considerations

The present study has been approved in Tabriz University of Medical Sciences (NO. IR.TBZMED.VCR.REC.1398.139).

## Results

### Studies characteristics

**Fig. 1:**
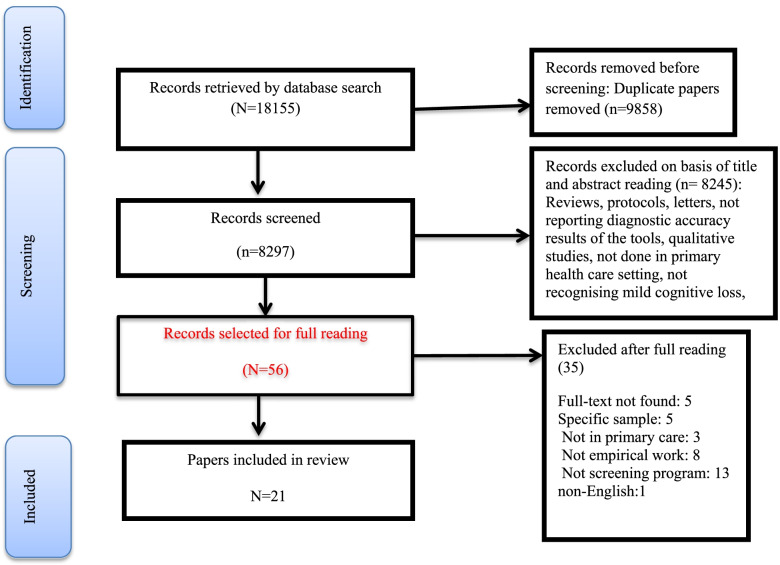
Study selection process

Systematic search identified 18,155 records, of which 9,858 articles were duplicates, and 8,245 records were not relevant which were excluded at initial screening of title and abstracts. After reviewing the title and abstract of the studies, 56 original articles were selected for the study. Of these, 35 studies were excluded because of not having eligible criteria. Finally twenty-one studies met the inclusion criteria for the systematic review and were included in the qualitative evaluation (Fig. 1). Characteristics of the studies were presented in Table 1, share of countries from the 21 final studies including Australia (*n* = 1) [18], China (*n *= 2) [19, 20], England (*n* = 1) [21], Germany (*n* = 3) [22–24], Greece (*n* = 2) [25, 26], Indonesia (*n* = 1) [27], Italy (*n* = 1) [28], Iran [29], Singapore (*n* = 1) [30], Portugal (*n* = 1) [31], Malaysia (*n* = 3) [32–34], Turkey (*n* = 1) [35], and USA (*n* = 3) [36–38] were studied.

**Table 1:** Study characteristics

ID	Sample Size	Country/income level	Screening tool	Age	Mode of delivery	Sensitivity and specificity
Arabi 2013	160	Malaysia/ upper-middle	EDQ and MMSE	65 >	Face-to-face or telephone interview with the patient and an informed person. The highest score was entered in the analysis	A score above 8 for EDQ and a score of 21 or lower for MMSE were considered as the criteria for diagnosing cognitive impairment
Arabi 2016	200	Malaysia/ upper-middle	EDQ and MMSE	60	Face-to-face and telephone interviews with patients and knowledgeable people around	A score of 6 was considered for EDQ for 95.5% sensitivity and 84.2% for specificity and a score of 21 for MMSE was considered for dementia diagnosis
Brodaty 2016 [20]	1717	Australia/ high	GPCOG, MMSE, CAMCOG	75	Nurses examined patients with MMSE and GPCOG, and then specialists used CAMCOG and GDS to evaluate patients at a later stage	A score below 5 indicates cognitive impairment and a score above 8 indicates a low probability of developing cognitive impairment. A score of 5 to 8 indicates an uncertain situation that requires assessment with GPCOF. A score of 11/10 out of 15 meant cognitive impairment in males. A score of 23 out of 30 is also considered cognitive impairment for MMSE
Chan 2016 [31]	309	Singapore/ high	AD8, MMSE, MOCA	60	Screening was done in two stages. In the first stage, psychiatrists evaluated the patient and in the second stage, a panel of specialists	AD8 with a cut-off point of 4.8 with a mean area below the curve of 0.97 and a sensitivity of 0.91 and a characteristic of 0.91 showed the best detection accuracy
Eichler 2015 [26]	4046	Germany/ high	DemTec vs MMSE	70	Demtec was first used by a GP for screening, then MMSE was used	The MMSE score was used to classify the cognitive impairment 27–30%: No disorder, 20–26%: Mild disorder, 10–19% Moderate disorder, 0–19%: Severe cognitive impairment
Grober 2014 [38]	112	US/ high	MMSE, screening	65	Screening was done in two stages. First, the individual's cognitive status was assessed using MMSE. If the individual's cognitive impairment was not diagnosed, a complete assessment using pFCSRT + IR was used	An MMSE score of 23 or higher was considered
Grober 2017 [39]	563	US/ high	IQCODE-	65	In the first stage, knowledgeable people completed the IQCODE short questionnaire. If a person was diagnosed with severe dementia, a complete evaluation was performed with pFCSRT-IR and DSM-IV	The diagnosis of cognitive impairment and dementia was made based on the opinion of a psychiatrist and a geriatrician
Grober 2019 [37]	257	US/ high	IQCOD and pFCSRT		Screening is done in two ways based on the patient and based on the informed caregiver and also in two stages. Initially, IQCOD was used if the person was accompanied and PBS was used if they were not. If the person is positive in the first stage, he / she enters the second stage and is evaluated through pFCSRT. If the person is negative, the screening will be repeated one year later. If the result of the second stage is positive, treatment is started and follow-up is done. If it is negative, the screening is repeated one year later	The decision criterion based on IQCODE: greater than equal to 3.5. The decision criterion in BPS is: MIS < 5 or AF < 9. In the second stage, if FR < 25 or TR < 46, the person is recognized as positive
Koc Okudur 2019 [36]	357	Turkey/ upper-middle	MMSE and RCS-T	60	First the evaluation was done based on MMSE and then the complete evaluation was done with RCS-T. The evaluation was performed by a general practitioner	The RCS score is from 0 to 10 A score of less than 4 was considered for the diagnosis of Alzheimer's and less than 6 for the diagnosis of mild cognitive impairment
Larner 2018 [23]	676	England/high	MMSE, MOCA and DSM-IV	65	The evaluation was based on MMSE and MOCA and in the second stage, based on the opinion of experts, the cognitive status of individuals was determined through DSM-V	Intersection points 24 and 26 were considered for MMSE
Latraki 2017 [28]	319	Greece/ high	TYM GPCog, MMSE	60–89	First TYM then GPCOG was used to diagnose cognitive impairment	TYM: The highest score is equal to 50, with two cutting points of 39.38 or 36.35. GPCOG: The maximum score is 9, with a cut-off point of 7 MMSE: 23 out of 30 as cutting point
Pandhita 2019	212	Indonesia/ upper-middle	CERAD	60	Used a decision tree model to identify cognitive impairment. The information entered in the model is based on CERAD, OLB and fast cognition assessment such as clock drawing, verbal test	Based on standard scores of WAHYU, VFT, SMC tests
Petrazzuoli 2014 [30]	121	Italy/ high	AQT and MMSE	45–90	Screening during routine referral to primary care was performed by a general practitioner	AQT sets different range for diagnosis (in seconds) for different age groups. No cutting point was reported for MMSE
Salami 2019 [29]	114	Iran	MMSE and TYM	80	Participants passed a physical examination and completed forms of the MMSE and TYM tests	The MMSE test had AUC = 0.991, sensitivity = 0.90 and specificity = 0.96,
Shaaban 2013 [35]	49	Malaysia/ upper-middle	M_RUDAS, MMSE, ECAQ	65	Screening by a family physician and a trained expert using M_RUDAS, M_MMSE and M_ECAQ. Clinical interview was conducted by a psychiatrist using DSM IV	MMSE: Cutting point 17 and less ECAQ: Cutting point 5/10 and less M-RUDAS: > 23
Stein 2015 [24]	6619	Germany/ high	SMSE, MMSE	75	MMSE with 30 questions and SMSE with 6 questions have been used by general practitioners. Additional evaluation by psychiatrists using DSM – III – R, DSM – IV	ROC and AUC have been used as measurement accuracy criteria. Cutting point as follows MMSE < 24 SMMSE < 4
Teixeira 2017 [32]	436	Portuguese/ high	MMSE and Global -GDS +	65	Patients are evaluated by a general practitioner or nurse and then caregivers of dementia patients are evaluated	Patients were divided into six groups based on the GDS result: Very mild cognitive impairment / Mild disorder / Moderate disorder / Moderate to severe disorder / Very severe disorder
Thyrian 2016 [25]	1167	Germany/upper-middle	MMSE vs Neuro psychiatric Inventory (NPI)	70	Evaluation of patients using MMSE by general practitioners and evaluation of their psychological status by psychiatrists by NPI	Classification of patients based on MMSE: 20–30%: Irrelevant or mild 10–19%: Medium 0–9%: Severe cognitive impairment An NPI score above 5 indicates a diagnosis of cognitive impairment
Xue 2017 [22]	2731	China/ upper-middle	SIS, (MMSE)	60	Screening was performed by trained health workers	MMSE: For education 0 to 5:17, Education 6 to 10 years: 20, And higher education than 10:24 as the cutting point, The cut-off point for SIS was set to 4
Yang 2014 [21]	733	China/ upper-middle	MMSE, MOCA versus SE + MOCA	60	The initial assessment was performed by nurses and face to face. MMSE and MoCA were used in combination to diagnose cognitive impairment	Cut points for MMSE (85.2 sensitivity and 92.75 specification, Illiterate: 17 out of 18, Up to 6 classes: 20 out of 21, More than 6 classes: 24 out of 25, Cut points for MoCA, Illiterate: 13 out of 14, Up to 6 classes: 19 out of 20, More than 20 classes: 24 out of 25
Zaganas 2020 [27]	314	Greece/ high	MMSE	60–100	Interviews were conducted by a trained nurse. Psychological assessment was performed by a trained psychiatrist who assessed the cognitive status of patients for more than 2.5 h	The cut-off point for the Greek version of MMSE was 24.23

According to World Bank classification of countries by income [39], fourteen studies were conducted in high income countries (Australia, England, Germany, Greece, Italy, Singapore, Portugal, and USA) and seven studies were conducted in upper-middle and low income countries (China, Indonesia, Malaysia, Turkey and Iran).

The studies mainly examined the age groups of 60 years and older, but in one study, the age group of 45 to 90 years was recruited [28]. In total, the present studies had totally 21,196 sample sizes that were performed in the general population. Short screening tools were used in all of the studies. The most widely used tool was the Mini Mental Status Examination (MMSE). The possibility of cognitive impairment was examined, so that in 17 studies (85%) [18–24, 26, 28–36], MMSE was used as a reference or index test. Due to the fact that the purpose of this study was to evaluate screening programs in the primary care ward, all studies were performed in primary care centers or family physician office. Screening was performed by family physicians or nurses or health care workers, and those whose cognitive status was positive at the first level (cognitive impairment), were referred to the secondary level (specialist clinics or psychiatrists or hospitals).

### Screening tests

As an index test, all studies used short tools to diagnose cognitive disorders. MMSE were used in 14 studies [18–23, 25–30, 32–34, 38], General Practitioner Assessment of Cognition (GPCOG) in two studies [18, 26], Test Your Memory (TYM) in two study [26, 29], Early Dementia Questionnaire (EDQ) in two studies [32, 33], Ascertain Dementia 8-item (AD8) in one study [30], the Informant Questionnaire On Cognitive Decline in the Elderly (IQCODE) in one study [37], the Picture version of the Free and Cued Selective Reminding Test with Immediate Recall (pFCSRT + IR) in two studies [36, 38], Malay Version Rowland Universal Dementia Assessment Scale (M-RUDAS) in one study [24], a new screening method to support diagnosis of dementia (DemTect) in one study [34], and the Consortium to Establish a Registry for Alzheimer's Disease (CERAD) in one study [27]. Also, as a reference test, 10 studies have used the agreement of psychiatrists or geriatricians [20, 21, 23, 25, 27, 28, 31, 35–37], one study [19] used CAMCOG, eight studies used MMSE [20, 24, 26, 29, 30, 33, 34, 37] and two studies used MOCA [19, 30] (Table 1).

### EDQ and MMSE

The accuracy of EDQ diagnosis and its comparison with MMSE has been studied in two studies [32, 33]. In these studies, the sensitivity for EDQ was (0.669, 0.799) and the specificity was (0.477, 0.651). Positive and negative predictive values for EDQ were 23.5% and 93.2%, respectively. In one study, EDQ was compared to MMSE [32]. The prevalence of dementia was estimated 52.3% by using EDQ and 15.2% by using MMSE. Based on the findings of these two studies, EDQ has been introduced as a suitable alternative tool for MMSE for screening in primary care settings. Since this tool is tailored with the patients’ symptoms in a specific condition, so it has a high accuracy of diagnosis. Given the high negative predictive value of this test, the researchers believed that fewer cases of patients would be concealed from screening. Also, as this tool is more powerful than MMSE in diagnosing patients in the early stages of the disease, it has high power for detecting patients in early stage of cognitive disorders.

### GPCOG and MMSE

The comparison of these two tests has been done in only one study [18]. In this study, the mean area under the curve (AUC) for GPCOG and MMSE was estimated to be 0.92 and 0.91%, respectively. However, there were no statistically significant differences between the two parameters. The sensitivity of GPCOG at the cut-off point of 11/10 and the sensitivity of MMSE at the cut-off point of 24/23 were estimated to be 0.79 and 0.51, respectively, which was also statistically significant. Researchers have reported better performance for GPCOG than MMSE despite spending less time for interviewing.

### AD8, MMSE, and MOCA

The diagnostic features of the AD8, MMSE, and MOCA tools have been compared in a study [30] by using ROC curve. In order to evaluate the accuracy of diagnosis of these tools, a panel of experts has been used as the reference standard. Based on the findings, among people over 60 years with a cut-off point of 3.4, the sub-curve area criterion (AUC) for AD8 is equal to 0.97 with a 95% confidence interval (0.95—0.99), with sensitivity of 0.91, positive predictive value of 0.63, and negative predictive value of 0.97. For MOCA with a cut-off point of 16.17 AUC, sensitivity, specificity, positive predictive value and negative predictive value were 0.94 (0.92- 0.97), 0.84, 0.89, 0.56 and 0.97, respectively. The AD8 is superior to the MMSE and has similar performance to the MOCA. The AD8 showed similar performance among people over 75 years of age. In the Yang study [19], MMSE and MOCA were used among elderly population. Although the purpose of this study was not to compare the two tools, both instruments performed well in terms of evaluator agreement. In the Larner’s study, AUC of 0.64, sensitivity and specificity were reported 0.80 and 0.86, respectively, for MMSE (index test) compared to MOCA (reference test). Due to the low sensitivity of MMSE, researchers have not considered this tool suitable for use in screening in low prevalence areas for cognitive impairment and have introduced alternative tools such as MOCA with more efficiency. The researchers believed that, regardless of the cost of using MMSE and copyright considerations, it is not suitable for use in primary care in low prevalence conditions.

### SIS and MMSE

The Short Screening Tool (SIS) [20] was derived from the MMSE tool. The different cutting points for the sensitivity of SIS have been reported. The most suitable cutting point is three, which has the sensitivity equal to 0.86, the specificity of 0.87, and AUC 95% CI: 0.93 (0.89–0.97). Researchers have found good validity for the SIS and believed that the summary of the SIS reduces the interview time and it is suitable for use among illiterate elderly population.

### pFCSRT + IR and MMSE

Grobber [38] compared the diagnostic characteristics of two combined tools picture version of the Free and Cued Selective Reminding Test with Immediate Recall (pFCSRT) plus IR and *MMSE*. The AUC for pFCSRT + IR was greater than the MMSE (86% vs. 72%, P < 0.026). For diagnosis of dementia with the same specificity (81%), the sensitivity of MMSE was 48% (cut-off point less than 24) and the sensitivity of pFCSRT + AR was 70% (cut-off point less than 27). The sensitivity was reported 74% for both tests (cut point less than 28 for pFCSRT) and (cut point more than > 26) for MMSE. The specificity of pFCSRT was 75% and MMSE was 62%. The accuracy of pFCSRT was superior to MMSE. These tools take 10 to 15 min to be completed.

### Pooled estimation of diagnostic accuracy of MMSE test

**Fig. 2:**
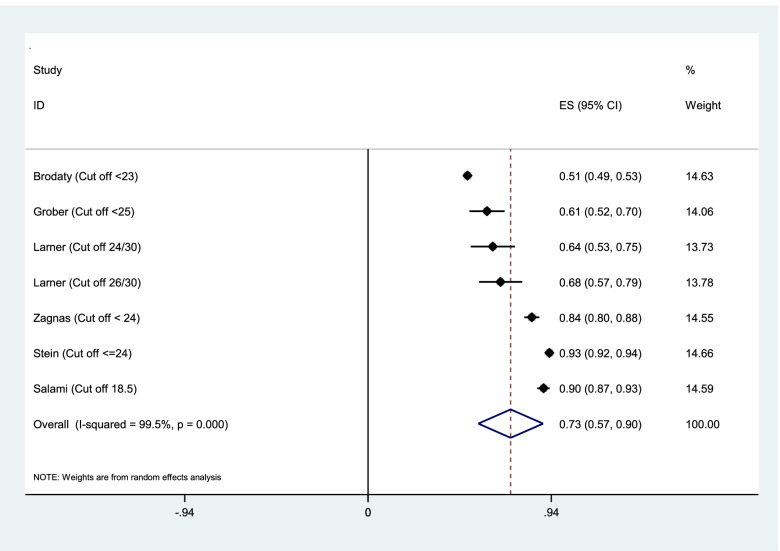
Results of aggregation of MMSE test sensitivity values in identifying cognitive disorders

**Fig. 3:**
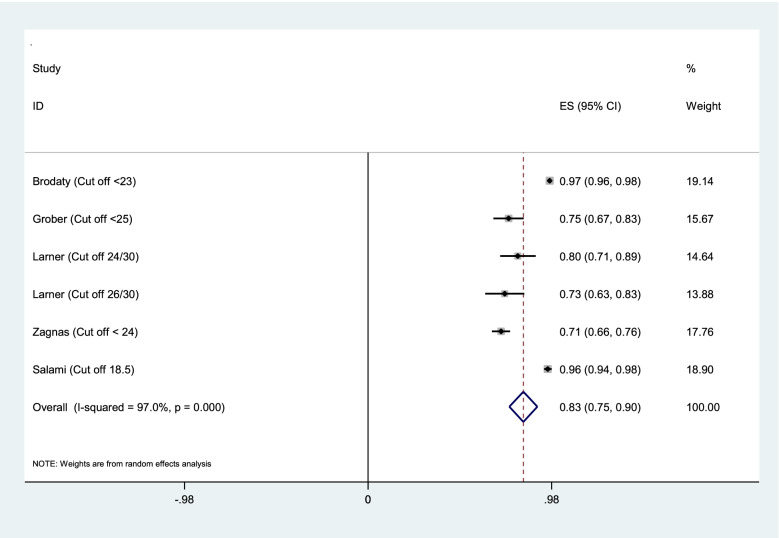
Results of aggregation of MMSE specificity feature values in identifying cognitive disorders

**Fig. 4:**
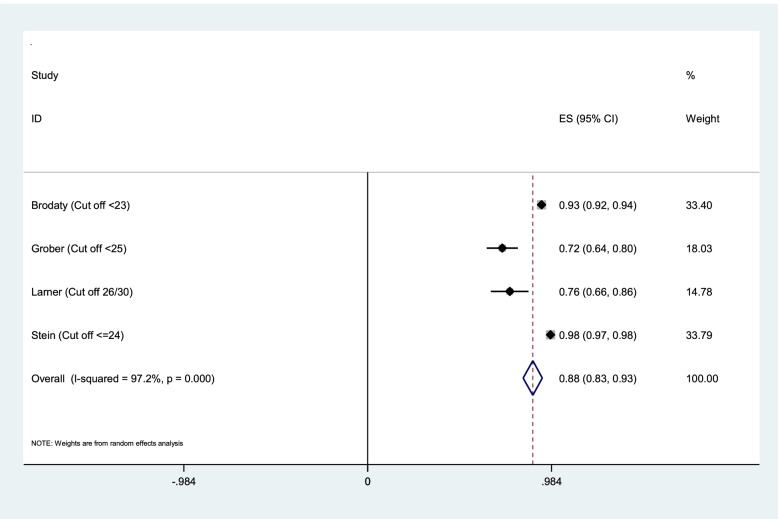
Results of aggregation of area under curve for MMSE test in identifying cognitive disorders

Aggregation of the values reported in seven studies for the sensitivity, specificity, and AUC of the MMSE test were used for meta-analysis. The cumulative sensitivity, specificity, and AUC analysis was conducted only for MMSE instrument. Due to the high heterogeneity in the studies, it was not possible to perform pooled analysis for all instruments. The diagnostic performance of the instruments used in the studies was systematically reviewed comparatively, the findings of which are presented in the following section. The random effect model for the MMSE showed the pooled sensitivity equal to 0.73 (95% CI 0.57–0.90) (Fig. 2), the pooled specificity equal to 0.83 (95% CI 0.75—0.90) (Fig. 3), and the pooled AUC 0.88 (95% CI 0.83–0.93) (Fig. 4).

**Fig. 5:**
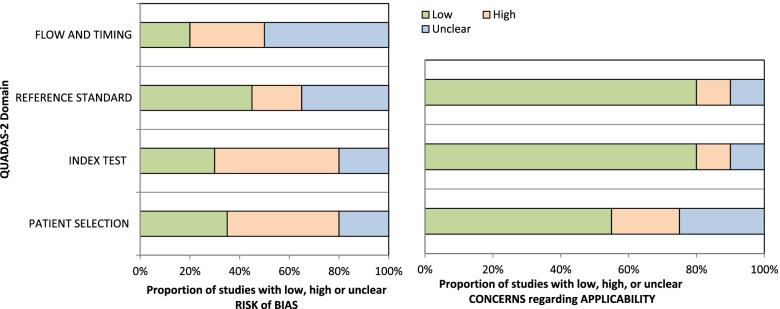
Risk of bias and concern about the applicability of each domain of quality assessment studies based on QUADAS2 tools

The risk of bias in the studies is shown in Table 2. Also, the risk of bias and concern about the applicability of each domain of quality assessment studies based on QUADAS2 tool were shown in Fig. 5. Kapa coefficient score was estimated 0.908 (*P* < 0.0001) indicating strong agreement between two screening researchers.

**Table 2:** Risk of bias in studies included in the systematic review using the QUADAS2 tool

Study	Risk of bias				Applicability concerns		
	Patient selection	Index test	Reference standard	Flow and timing	Patient selection	Index test	Reference standard
Brodaty [20]	LR	UR	HR	LR	LR	HR	LR
Zaganas [27]	UR	LR	LR	HR	LR	LR	LR
Arabi [33]	HR	HR	LR	UR	LR	LR	LR
Arabi 2016 [34]	LR	LR	UR	LR	LR	LR	LR
Chan [31]	UR	LR	LR	UR	HR	LR	LR
Eichler [26]	LR	HR	LR	UR	LR	LR	LR
Grober 2014 [38]	HR	LR	LR	UR	UR	LR	LR
Grober 2017 [39]	HR	HR	LR	UR	UR	LR	LR
Grober 2016 [37]	LR	HR	LR	UR	UR	LR	LR
Iatrakia 2017	HR	HR	HR	UR	UR	UR	LR
Okudur [36]	UR	HR	LR	UR	LR	LR	LR
Larner [23]	UR	HR	HR	LR	HR	LR	UR
Pandhita [27]	LR	HR	UN	LR	HR	HR	HR
Petrazzuoli [30]	LR	HR	HR	LR	LR	UN	LR
Salami 2019 [29]	HR	LR	UN	HR	HR	LR	UN
Shaaban [35]	HR	UN	UN	HR	LR	LR	LR
Stein	LR	UN	LR	HR	LR	LR	LR
Teixeira [32]	HR	LR	UN	HR	LR	LR	UR
Thyrian	HR	UN	UN	HR	UN	LR	LR
Xue [22]	HR	LR	UN	UN	LR	LR	HR
Yang [21]	HR	HR	UN	HR	UN	LR	LR

*Note: LR* Low Risk, *HR* High Risk, *UR* Unclear Risk

### Subgroup analysis

Table 3 shows the results based on the sensitivity, specificity, and AUC of MMSE according to subgroup analyses to explore the origin of the heterogeneity between the studies. The random-effects pooled estimation for sensitivity was 0.71 (95% CI 0.53–0.88; *p* < 0.001), for specificity was 0.81 (95% CI 0.67–0.95; *p* < 0.001), and for AUC was 0.73 (95% CI 0.67–0.80; *p* < 0.001) for participants aged 75 years and older. The higher random effect pooled estimation for sensitivity for the groups with respect to country’s income was for 0.91 low income countries (95% CI 0.89–0.94). The higher random effect pooled estimation for specificity was 0.97 (95% CI 0.96–0.97; *p* < 0.001) and for AUC was 0.97 (0.64–0.94; *p* < 0.001), respectively, for the groups with respect to sample size > 1000.

**Table 3:** Findings of subgroup analyses based on sensitivity, specificity, and AUC for MMSE

	No. of studies	Pooled Estimates [95% CI]	I^2^	p-Value for Heterogeneity	Tau-Squared
**Sensitivity**					
**Age** (year)					
≥ 75	3	0.71 (0.53–0.88)	99.8	< 0.001	0.67
< 75	3	0.76 (0.46–1.05)	94.9	< 0.001	0.03
**Sample size**					
> 1000	2	0.72 (0.30–1.13)	99.9	< 0.001	0.08
≤ 1000	5	0.74 (0.64–0.89)	93.2	< 0.001	0.01
**Country’s income**					
High income	5	0.56 (0.48–0.83)	98.1	< 0.001	0.0379
Low income	2	0.91 (0.89–0.94)	69.1	< 0.001	0.0003
**Specificity**					
**Age** (year)					
≥ 75	3	0.81 (0.67–0.95)	93.9	< 0.001	0.018
< 75	3	0.84 (0.58–1.09)	99.1	< 0.001	0.035
**Sample size**					
> 1000	2	0.97 (0.96–0.97)	0	< 0.001	0
≤ 1000	5	0.79(0.65–0.93)	96.7	< 0.001	0.023
**AUC**					
**Age** (year)					
≥ 75	5	0.73(0.67–0.80)	0	< 0.001	0
< 75	2	0.95 (0.90–1.00)	98.3	< 0.001	0.001
**Sample size**					
> 1000	2	0.97 (0.97–0.98)	98.3	< 0.001	0.01
≤ 1000	5	0.73 (0.65–0.86)	0	< 0.001	0
**Country’s income**					
High income	5	0.79 (0.64–0.94)	97.6	< 0.001	0.027
Low income	2	0.96 (0.94–0.98)	0	< 0.001	0

### Publication bias

**Fig. 6:**
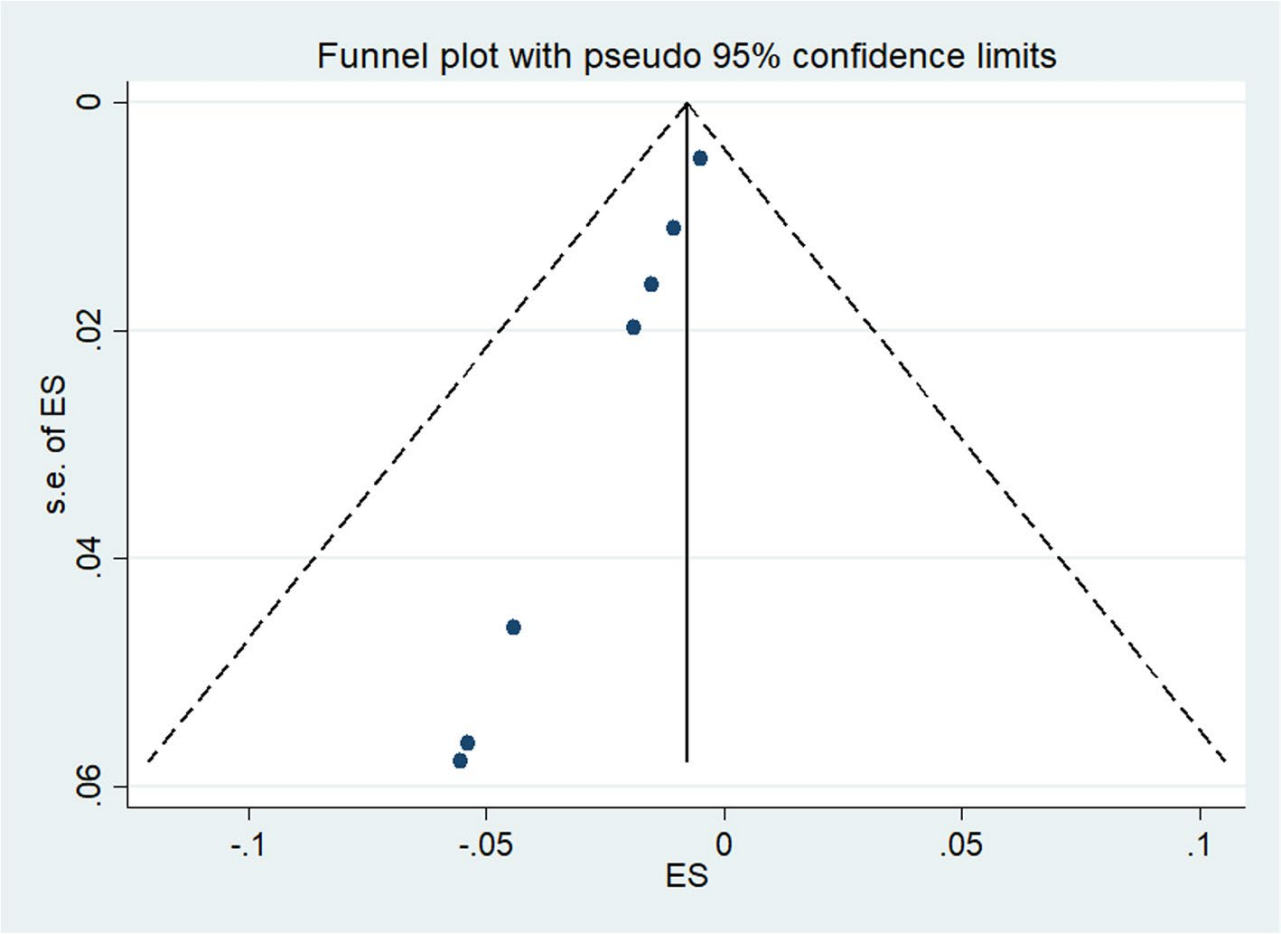
Funnel plot

Publication bias was highlighted and confirmed by funnel plots. The funnel plots in Fig. 6 testing publication within diagnostic accuracy of MMSE tool. The graphical results point to asymmetry with a majority of the studies clustering to the left of the mean. Large studies are shown at the top of the graph, and smaller studies are shown at the bottom.

## Discussion

The findings of the systematic review showed that the MMSE questionnaire is the most widely used tool and has been used as an indicator or reference test in most studies.

The findings of the present systematic review showed that there is insufficient evidence for community-based screening programs. The included final 21 studies in the systematic review also performed early detection of cognitive disorders on cross-sectional samples of the population and reported the accuracy of diagnosis of these tools. Of the 21 final studies, two studies [24, 27] recommended routine screening for cognitive disorders and three studies recommended against screening [19, 31, 36] that have pointed to the inability to implement community based screening, especially in low-income countries. Some substantial barriers of screening for cognitive disorders in low-income countries were highlighted such as limited resources for serving large population, insufficient training, and shortage of general physicians [19]. Another issues like living of most of older adults in remote rural areas or urban areas without having access the centers where offer routine screening tests [40]. These factors, along with other epidemiological and social factors like low educational level, low socio-economic status of older population, time and financial constraints, diagnostic uncertainty, stigma [35], and access of such people to health care centers contribute to the pause and challenging of screening programs in low income countries [5, 41] However, Koch et al. [35] in a rapid appraisal of barriers to the diagnosis of cognitive disorders and dementia stated that health care systems were accountable for the several mentioned barriers [42]. Eichler [24] and Pandahita [27] agreements for performing routine screening were the high percentage of undiagnosed patients in primary care settings and also the fact that the proposed screening test did not provide enough information about the feasibility of screening. Therefore, these two studies would not be recognized sufficient evidence for screening cognitive disorders. The findings are in line with the recommendations of the US Preventive Services Committee Task Force (USPSTF) in 2003, 2011, 2014 and most recently in 2020. The committee believes that there was no evidence to prove the screening program could improve the current care process [12]. The Alzheimer's Association of the United States cites this evidence and recommends the inclusion of an early detection program for cognitive disorders in the annual geriatric visits [5, 43]. Iliffe et al. [43] stated that they were not able to identify an advantage for routine screening test, but they considered the possibility of early detection in primary care. Therefore, the program for diagnosing cognitive disorders is beyond the informal observation by a physician and is an ongoing process that is diagnosed during various stages of senile disorder. Counselling and interviewing before and after the diagnosis of the disorder is an important part of the diagnosis process and the use of caregivers and elderly people would be effective in diagnosing the disease [43]. The National Institute for Clinical Excellence (NICE) and the UK National Health System's advisory did not consider routine screening to be cost-effectiveness in their recommendations in 2006.

More than 12 different tools have been used in the final studies. MMSE tool is the most widely used and common tool in this field. Comparison of instruments showed that IQCODE, GPCOG, AD8, MOCA, PFCSRT + IR and EDQ instruments had detection power equal to or higher than MMSE. Even the MMSE short tool had good diagnostic performance. The present finding shows that the above tools can replace MMSE in the diagnosis of cognitive disorders and dementia. In addition, MMSE because of being long, not free and is biased towards the literacy level of the participants, the Alzheimer's Association has introduced six criteria for selecting the right tool, including evaluation time of less than 5 min, validation evidence in primary care, usability by non-medical staff, appropriate psychometric properties, insensitivity to literacy, language and culture bias, and it’s free availability. The Alzheimer's Association based on the findings of the previously published systematic review studies [44–47] showed appropriate tools for assessing patients' cognition, including GPCOG, Mini-Cog, and MIS, and interviews with IQCODE, AD8, and GPCOG caregivers. Our systematic review findings are also in line with the recommendations of the Alzheimer's Association. MOCA, IQCODE, GPCOG and MMSE instruments have also been validated in Iran [29, 48, 49], but participants were recruited from the general population setting rather than the primary care units. Consistent with our study, a review study on brief cognitive screening instruments found that MMSE is the most frequently used cognitive screening tool in the community and primary care. The study also highlighted that mini cognition (Mini-cog), memory impairment screen (MIS), and the general practitioner assessment of cognition (GPCOG) were beneficial in primary care setting and recommended for use [47]. Based on the findings, practicality, psychometric properties of instruments, validation in a community, general population, or referring people for primary care setting, as well as utility, efficacy, and administration time were major criteria for implementing the cognitive screening instruments in primary care and community programs especially in low income countries.

### Limitations

The available studies were carried out in the variety of high and middle income countries. There was no study in low level country to clarify the advantages or disadvantages of screening programs in these countries. Overall, additional researches are needed to identify the best screening tool in low income countries.

## Conclusion

There was insufficient evidence for routine and general screening to identify cognitive disorders. However, due to the high incidence of undiagnosed patients and the benefits of early diagnosis in caregiver management, the integration of early diagnosis into annual or periodic geriatric care programs has been used in most high-income countries. The use of non-medical staff in the initial assessment can be suggested as a suitable option, especially in countries that face a shortage of medical staff. Although MMSE is the most widely used diagnostic tool, according to the current systematic review, MOCA, GPCOG and MIS tools can be used to evaluate patients and IQCODE, AD8 and GPCOG tools can be used to evaluate their caregivers with equal or better performance than MMSE.

## Data Availability

The data collection tools and datasets generated and/or analyzed during the current study are available from the corresponding author on reasonable request.

## Abbreviations

AQT: A Quick Test of Cognitive Speed,
RCS-T: Rapid Recognition Screening
SIS: Six-Item Screener
PRISMA: Preferred report items for systematic review and meta-analysis
QUADAS: Quality Assessment of Diagnostic Accuracy Studies-2
AWV: Annual wellness visit
MMSE: Mini–mental state examination
GPCOG: General practitioner assessment of cognition
AD8: Ascertain dementia 8-item
TYM: Test your memory
EDQ: Early Dementia Questionnaire
IQCODE, pFCSRT + IR: The picture version of the Free and Cued Selective Reminding Test with Immediate
M-RUDAS: The Recall Malay Version Rowland Universal Dementia Assessment Scale
DemTect: A new screening method to support diagnosis of dementia
